# A one-dimensional coordination polymer constructed from isatine-3-oximate and sodium

**DOI:** 10.1107/S1600536811018290

**Published:** 2011-05-25

**Authors:** Bianca Barreto Martins, Leandro Bresolin, Vanessa Santana Carratu, Mariana Boneberger Behm, Adriano Bof de Oliveira

**Affiliations:** a Escola de Química e Alimentos, Universidade Federal do Rio Grande, Av. Itália km 08, Campus Carreiros, 96201-900 Rio Grande, RS, Brazil; b Departamento de Química, Universidade Federal de Santa Maria, Av. Roraima, Campus, 97105-900 Santa Maria, RS, Brazil; c Departamento de Química, Universidade Federal de Sergipe, Av. Marechal Rondon s/n, Campus, 49100-000 São Cristóvão, SE, Brazil

## Abstract

The reaction of hydroxyl­amine hydro­chloride with isatin in ethanol, catalysed with HCl and neutralized with Na_2_CO_3_, yielded the one-dimensional coordination polymer, *catena*-poly[[[aqua­sodium]-di-μ-aqua-[aqua­sodium]-bis­(μ-2-oxoindoline-2,3-dione 3-oximato)] tetra­kis­(oxoindoline-2,3-dione 3-oxime)], {[Na(C_8_H_5_N_2_O_2_)(H_2_O)_2_]·2C_8_H_6_N_2_O_2_}_*n*_. The Na^I^ atom has a six-coordinate distorted-octa­hedral environment. Isatine-3-oximate O atoms and water mol­ecules bridge adjacent Na atoms, forming a one-dimensional polymeric structure parallel to [100]. Each isatine-3-oxime dimerizes through N—H⋯O interactions and in addition each oxime is linked to a coordination polymer. Thus, coordination polymers are linked by O—H⋯O and O—H⋯N interactions from isatine-3-oxime dimers, building a two-dimensional network parallel to [110].

## Related literature

For the pharmacological and biological properties of oxime derivatives, see: Chafeev *et al.* (2008 ▶). For the preparation and characterization of some metal complexes of isatine-3-oxime, see: Hudák & Košturiak (1999 ▶).
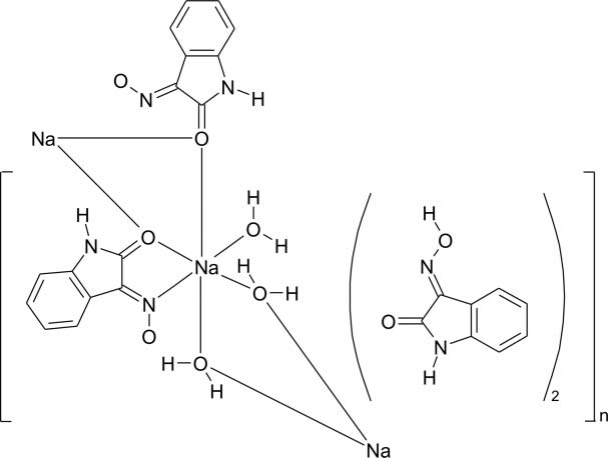


## Experimental

### 

#### Crystal data

[Na(C_8_H_5_N_2_O_2_)(H_2_O)_2_]·2C_8_H_6_N_2_O_2_
*M* *_r_* = 544.45
Triclinic, 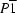

*a* = 7.2987 (3) Å
*b* = 11.9269 (5) Å
*c* = 15.0756 (6) Å
α = 95.369 (2)°
β = 102.871 (2)°
γ = 102.631 (2)°
*V* = 1234.36 (9) Å^3^
*Z* = 2
Mo *K*α radiation
μ = 0.13 mm^−1^
*T* = 293 K
0.74 × 0.23 × 0.18 mm

#### Data collection

Bruker CCD X8 APEXII diffractometer
Absorption correction: multi-scan (*SADABS*; Bruker, 2003 ▶) *T*_min_ = 0.912, *T*_max_ = 0.978
25159 measured reflections
7175 independent reflections
4225 reflections with *I* > 2σ(*I*)
*R*_int_ = 0.029

#### Refinement

*R*[*F* ^2^ > 2σ(*F* ^2^)] = 0.049
*wR*(*F* ^2^) = 0.135
*S* = 1.04
7175 reflections
388 parameters
H atoms treated by a mixture of independent and constrained refinement
Δρ_max_ = 0.34 e Å^−3^
Δρ_min_ = −0.35 e Å^−3^

### 

Data collection: *SMART* (Bruker, 2003 ▶); cell refinement: *SAINT* (Bruker, 2003 ▶); data reduction: *SAINT*; program(s) used to solve structure: *SHELXS97* (Sheldrick, 2008 ▶); program(s) used to refine structure: *SHELXL97* (Sheldrick, 2008 ▶); molecular graphics: *DIAMOND* (Brandenburg, 2006 ▶); software used to prepare material for publication: *SHELXL97*.

## Supplementary Material

Crystal structure: contains datablocks I, global. DOI: 10.1107/S1600536811018290/bt5520sup1.cif

Supplementary material file. DOI: 10.1107/S1600536811018290/bt5520Isup2.mol

Structure factors: contains datablocks I. DOI: 10.1107/S1600536811018290/bt5520Isup3.hkl

Additional supplementary materials:  crystallographic information; 3D view; checkCIF report

## Figures and Tables

**Table 1:** Hydrogen-bond geometry (Å, °)

*D*—H⋯*A*	*D*—H	H⋯*A*	*D*⋯*A*	*D*—H⋯*A*
N21—H21⋯O21^i^	0.87 (2)	1.97 (2)	2.846 (5)	175 (2)
O22—H22⋯O12	0.94 (2)	1.70 (2)	2.633 (3)	171 (2)
O22—H22⋯N12	0.94 (2)	2.45 (2)	3.226 (4)	140 (2)

Symmetry code: (i) 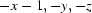.

**Fig. 1.**
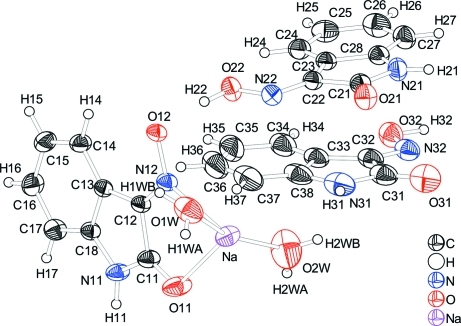
: The molecular structure of [Na(C8N2O2H5)(H2O)2](C8N2O2H6)2 with 40% probability displacement ellipsoids for non-H atoms.

**Fig. 2.**
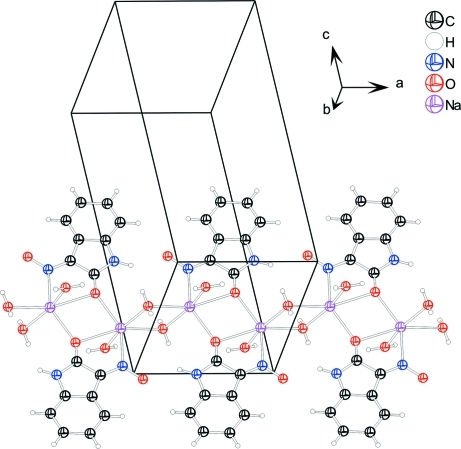
: The one-dimensional sodium(I) coordination polymer along a axis. The graphical representation is simplified for clarity.

## supplementary crystallographic information

### Comment

Oxime derivatives such as isatine-3-oxime have a wide range of properties. For example, they modulate ion flux through a voltage-dependent sodium channel in a mammal. Acting as small molecule sodium channel blockers, these compounds are used intreating diseases or conditions such as hypercholesterolemia and cancer (Chafeev *et al.*, 2008). As part of our interest in the study of oxime derivatives, we report herein the crystal structure of [Na(C_8_N_2_O_2_H_5_)(H_2_O)_2_](C_8_N_2_O_2_H_6_)_2_. Crystallographically independent, the structure contains two protonated isatine-3-oxime, two water molecules, one isatine-3-oximate and one sodium(I). The sodium cation has a six-coordinate distorted octahedral environment: one ON-donor bidentate oximate and two water molecules are crystallographically independent. One O-donor monodentate oximate, symmetry generated^ii^, and one symmetry generated water molecule^i^ complete the coordination sphere. The polymeric structure is build one side with two bridging water molecules, one crystallographicaly independent and one symmetry generated^i^ and another side with one bridging oxygen atom from a crystallographically independent oximate and one oxygen atom from a symmetry generated^ii^ oximate. These O-donor bridging atoms form a 1-D zigzag chain of Na complexes in solid state. For the sodium(I) coordination sphere the metal-donor distances [Å] are: Na—O11 = 2,5721 (14), Na—O11^ii^ = 2,3281 (14), Na—O2W = 2,361 (2), Na—O1W = 2,4117 (17), Na—O1W^i^ = 2,485 (18), Na—N12 = 2,4906 (15). Selected angles (°) are: O1W^i^—Na—O11 = 158,74 (6), O2W—Na—O1W = 162,92 (8), O11^ii^—Na—N12 = 141,63 (6) and build a distorced octahedra. Symmetry codes: (i)*-x,-y + 1,-z*; (ii)*-x + 1,-y + 1,-z*.

### Experimental

Starting materials were commercially available and were used without further purification. The synthesis was adapted from a procedure reported previously (Chafeev *et al.*, 2008; Hudák & Košturiak, 1999). The hydrochloric acid catalyzed reaction of isatin (8,83 mmol) and hydroxylamine hydrochloride (8,83 mmol) in ethanol (50 ml) was refluxed for 6 h and neutralized with a 10% solution of sodium carbonate in water (50 ml). After cooling and filtering, crystals suitable for X-ray diffraction were obtained.

### Crystal data

**Table:**

[Na(C_8_H_5_N_2_O_2_)(H_2_O)_2_]·2C_8_H_6_N_2_O_2_	*Z* = 2
*M**_r_* = 544.45	*F*(000) = 564
Triclinic, *P*1	*D*_x_ = 1.465 Mg m^−^^3^
Hall symbol: -P 1	Melting point: 507 K
*a* = 7.2987 (3) Å	Mo *K*α radiation, λ = 0.71073 Å
*b* = 11.9269 (5) Å	Cell parameters from 25159 reflections
*c* = 15.0756 (6) Å	θ = 2.1–30.1°
α = 95.369 (2)°	µ = 0.13 mm^−^^1^
β = 102.871 (2)°	*T* = 293 K
γ = 102.631 (2)°	Block, yellow
*V* = 1234.36 (9) Å^3^	0.74 × 0.23 × 0.18 mm

### Data collection

**Table:**

Bruker CCD X8 APEXII diffractometer	7175 independent reflections
Radiation source: fine-focus sealed tube, CCD area detector	4225 reflections with *I* > 2σ(*I*)
graphite	*R*_int_ = 0.029
φ and ω scans	θ_max_ = 30.1°, θ_min_ = 2.1°
Absorption correction: multi-scan (*SADABS*; Bruker, 2003)	*h* = −8→10
*T*_min_ = 0.912, *T*_max_ = 0.978	*k* = −16→16
25159 measured reflections	*l* = −21→20

### Refinement

**Table:**

Refinement on *F*^2^	Primary atom site location: structure-invariant direct methods
Least-squares matrix: full	Secondary atom site location: difference Fourier map
*R*[*F*^2^ > 2σ(*F*^2^)] = 0.049	Hydrogen site location: inferred from neighbouring sites
*wR*(*F*^2^) = 0.135	H atoms treated by a mixture of independent and constrained refinement
*S* = 1.04	*w* = 1/[σ^2^(*F*_o_^2^) + (0.0563*P*)^2^ + 0.222*P*] where *P* = (*F*_o_^2^ + 2*F*_c_^2^)/3
7175 reflections	(Δ/σ)_max_ = 0.002
388 parameters	Δρ_max_ = 0.34 e Å^−^^3^
0 restraints	Δρ_min_ = −0.35 e Å^−^^3^

### Special details

**Table:**

Geometry. All s.u.'s (except the s.u. in the dihedral angle between two l.s. planes) are estimated using the full covariance matrix. The cell s.u.'s are taken into account individually in the estimation of s.u.'s in distances, angles and torsion angles; correlations between s.u.'s in cell parameters are only used when they are defined by crystal symmetry. An approximate (isotropic) treatment of cell s.u.'s is used for estimating s.u.'s involving l.s. planes.
Refinement. Refinement of *F*^2^ against ALL reflections. The weighted *R*-factor *wR* and goodness of fit *S* are based on *F*^2^, conventional *R*-factors *R* are based on *F*, with *F* set to zero for negative *F*^2^. The threshold expression of *F*^2^ > 2σ(*F*^2^) is used only for calculating *R*-factors(gt) *etc*. and is not relevant to the choice of reflections for refinement. *R*-factors based on *F*^2^ are statistically about twice as large as those based on *F*, and *R*- factors based on ALL data will be even larger.

### Fractional atomic coordinates and isotropic or equivalent isotropic displacement parameters (Å^2^)

**Table:**

	*x*	*y*	*z*	*U*_iso_*/*U*_eq_	
O1W	0.0994 (2)	0.63158 (15)	0.01259 (17)	0.0687 (5)	
O2W	0.3016 (4)	0.28216 (19)	0.02320 (19)	0.1066 (8)	
H1WA	0.130 (5)	0.663 (3)	−0.029 (3)	0.129 (15)*	
H1WB	0.119 (6)	0.683 (3)	0.063 (3)	0.143 (16)*	
H2WB	0.198 (5)	0.225 (3)	−0.008 (2)	0.104 (11)*	
H11	0.940 (3)	0.5518 (17)	0.2221 (14)	0.058 (6)*	
H21	−0.432 (3)	−0.0502 (18)	0.0682 (15)	0.058 (6)*	
H22	0.101 (3)	0.409 (2)	0.2678 (15)	0.072 (7)*	
H31	0.157 (3)	0.100 (2)	0.0853 (17)	0.071 (7)*	
H32	0.264 (4)	−0.314 (3)	0.283 (2)	0.109 (10)*	
H2WA	0.412 (6)	0.258 (3)	0.023 (3)	0.157 (15)*	
Na	0.24999 (10)	0.47107 (6)	0.02804 (5)	0.04703 (19)	
C11	0.6536 (2)	0.53381 (15)	0.18198 (12)	0.0393 (4)	
C12	0.5139 (2)	0.54630 (13)	0.23926 (11)	0.0322 (3)	
C13	0.6261 (2)	0.57694 (13)	0.33425 (10)	0.0307 (3)	
C14	0.5812 (2)	0.60035 (15)	0.41768 (11)	0.0405 (4)	
H14	0.4542	0.5980	0.4200	0.049*	
C15	0.7296 (3)	0.62728 (16)	0.49741 (12)	0.0474 (4)	
H15	0.7017	0.6430	0.5539	0.057*	
C16	0.9194 (3)	0.63120 (17)	0.49426 (12)	0.0473 (4)	
H16	1.0167	0.6498	0.5487	0.057*	
C17	0.9670 (2)	0.60815 (16)	0.41194 (12)	0.0432 (4)	
H17	1.0943	0.6110	0.4100	0.052*	
C18	0.8188 (2)	0.58075 (13)	0.33264 (11)	0.0328 (3)	
N11	0.83052 (19)	0.55582 (13)	0.24153 (10)	0.0405 (3)	
N12	0.33205 (18)	0.52849 (12)	0.19803 (9)	0.0358 (3)	
O11	0.61709 (18)	0.50974 (14)	0.09817 (9)	0.0622 (4)	
O12	0.21617 (15)	0.54444 (9)	0.25488 (8)	0.0371 (3)	
C21	−0.3048 (2)	0.11666 (15)	0.10061 (13)	0.0418 (4)	
C22	−0.1543 (2)	0.17741 (14)	0.18624 (12)	0.0374 (4)	
C23	−0.1264 (2)	0.09011 (14)	0.24606 (12)	0.0378 (4)	
C24	−0.0120 (3)	0.09265 (16)	0.33278 (13)	0.0453 (4)	
H24	0.0701	0.1619	0.3660	0.054*	
C25	−0.0219 (3)	−0.01047 (18)	0.36947 (14)	0.0530 (5)	
H25	0.0546	−0.0103	0.4278	0.064*	
C26	−0.1445 (3)	−0.11327 (18)	0.32007 (16)	0.0592 (5)	
H26	−0.1499	−0.1811	0.3463	0.071*	
C27	−0.2593 (3)	−0.11803 (16)	0.23284 (15)	0.0522 (5)	
H27	−0.3406	−0.1877	0.1999	0.063*	
C28	−0.2488 (2)	−0.01592 (14)	0.19665 (12)	0.0401 (4)	
C31	0.1466 (3)	−0.05497 (19)	0.11845 (18)	0.0596 (6)	
C32	0.2434 (3)	−0.09228 (16)	0.20606 (15)	0.0520 (5)	
C33	0.3592 (3)	0.01207 (16)	0.26774 (16)	0.0527 (5)	
C34	0.4738 (3)	0.03367 (19)	0.35712 (19)	0.0662 (6)	
H34	0.4890	−0.0271	0.3902	0.079*	
C35	0.5659 (3)	0.1486 (2)	0.3964 (2)	0.0780 (8)	
H35	0.6428	0.1653	0.4565	0.094*	
C36	0.5420 (4)	0.2381 (2)	0.3453 (3)	0.0875 (9)	
H36	0.6042	0.3143	0.3723	0.105*	
C37	0.4292 (4)	0.2177 (2)	0.2559 (2)	0.0790 (8)	
H37	0.4154	0.2784	0.2226	0.095*	
C38	0.3383 (3)	0.10472 (17)	0.2184 (2)	0.0658 (7)	
N21	−0.3501 (2)	0.00296 (13)	0.11123 (11)	0.0456 (4)	
N22	−0.0782 (2)	0.28682 (12)	0.19124 (10)	0.0413 (3)	
N31	0.2124 (3)	0.06162 (17)	0.12979 (17)	0.0685 (5)	
N32	0.2115 (2)	−0.20215 (13)	0.20959 (12)	0.0518 (4)	
O21	−0.37599 (19)	0.15939 (11)	0.03390 (9)	0.0522 (3)	
O22	0.05472 (18)	0.33089 (11)	0.27387 (9)	0.0472 (3)	
O31	0.0319 (3)	−0.11626 (16)	0.05048 (13)	0.0772 (5)	
O32	0.3051 (2)	−0.22606 (12)	0.29317 (10)	0.0575 (4)	

### Atomic displacement parameters (Å^2^)

**Table:**

	*U*^11^	*U*^22^	*U*^33^	*U*^12^	*U*^13^	*U*^23^
O1W	0.0589 (9)	0.0549 (9)	0.1021 (15)	0.0188 (7)	0.0294 (10)	0.0270 (10)
O2W	0.0800 (14)	0.0787 (14)	0.154 (2)	0.0381 (12)	0.0035 (14)	0.0028 (13)
Na	0.0443 (4)	0.0630 (5)	0.0378 (4)	0.0207 (3)	0.0113 (3)	0.0064 (3)
C11	0.0309 (8)	0.0550 (10)	0.0342 (9)	0.0132 (7)	0.0100 (7)	0.0075 (8)
C12	0.0262 (7)	0.0374 (8)	0.0347 (8)	0.0082 (6)	0.0102 (6)	0.0063 (7)
C13	0.0262 (7)	0.0334 (8)	0.0329 (8)	0.0075 (6)	0.0076 (6)	0.0054 (6)
C14	0.0317 (8)	0.0530 (10)	0.0376 (9)	0.0104 (7)	0.0116 (7)	0.0033 (8)
C15	0.0464 (10)	0.0597 (11)	0.0331 (9)	0.0094 (8)	0.0108 (8)	−0.0002 (8)
C16	0.0408 (9)	0.0604 (12)	0.0340 (9)	0.0107 (8)	0.0001 (7)	0.0010 (8)
C17	0.0288 (8)	0.0564 (11)	0.0432 (10)	0.0130 (7)	0.0044 (7)	0.0064 (8)
C18	0.0278 (7)	0.0371 (8)	0.0346 (9)	0.0095 (6)	0.0081 (6)	0.0063 (7)
N11	0.0269 (7)	0.0619 (9)	0.0366 (8)	0.0163 (6)	0.0111 (6)	0.0060 (7)
N12	0.0275 (6)	0.0435 (8)	0.0380 (8)	0.0098 (5)	0.0099 (6)	0.0069 (6)
O11	0.0414 (7)	0.1127 (12)	0.0331 (7)	0.0220 (7)	0.0103 (6)	0.0050 (7)
O12	0.0284 (5)	0.0396 (6)	0.0454 (7)	0.0076 (4)	0.0150 (5)	0.0039 (5)
C21	0.0382 (9)	0.0400 (9)	0.0480 (11)	0.0101 (7)	0.0132 (8)	0.0045 (8)
C22	0.0366 (8)	0.0348 (9)	0.0428 (9)	0.0083 (7)	0.0148 (7)	0.0053 (7)
C23	0.0360 (8)	0.0373 (9)	0.0438 (10)	0.0096 (7)	0.0168 (7)	0.0063 (7)
C24	0.0440 (10)	0.0463 (10)	0.0483 (11)	0.0109 (8)	0.0171 (8)	0.0078 (8)
C25	0.0554 (11)	0.0605 (12)	0.0526 (12)	0.0215 (10)	0.0201 (9)	0.0222 (10)
C26	0.0672 (13)	0.0481 (12)	0.0761 (15)	0.0221 (10)	0.0308 (12)	0.0273 (11)
C27	0.0545 (11)	0.0379 (10)	0.0676 (13)	0.0108 (8)	0.0220 (10)	0.0097 (9)
C28	0.0379 (9)	0.0372 (9)	0.0484 (10)	0.0102 (7)	0.0171 (8)	0.0052 (7)
C31	0.0543 (12)	0.0544 (13)	0.0858 (17)	0.0220 (10)	0.0372 (12)	0.0205 (12)
C32	0.0481 (10)	0.0448 (11)	0.0757 (14)	0.0168 (8)	0.0330 (10)	0.0160 (10)
C33	0.0461 (10)	0.0437 (10)	0.0800 (15)	0.0157 (8)	0.0342 (11)	0.0123 (10)
C34	0.0555 (12)	0.0521 (12)	0.1007 (19)	0.0148 (10)	0.0384 (13)	0.0098 (12)
C35	0.0565 (13)	0.0603 (15)	0.114 (2)	0.0060 (11)	0.0333 (14)	−0.0109 (14)
C36	0.0672 (16)	0.0467 (14)	0.155 (3)	0.0080 (11)	0.0546 (19)	−0.0051 (16)
C37	0.0702 (16)	0.0471 (13)	0.138 (3)	0.0199 (11)	0.0569 (17)	0.0200 (15)
C38	0.0596 (13)	0.0445 (11)	0.117 (2)	0.0228 (10)	0.0553 (14)	0.0232 (12)
N21	0.0433 (8)	0.0373 (8)	0.0513 (10)	0.0052 (7)	0.0095 (7)	0.0003 (7)
N22	0.0384 (7)	0.0405 (8)	0.0435 (8)	0.0068 (6)	0.0108 (6)	0.0048 (6)
N31	0.0694 (12)	0.0609 (12)	0.0961 (17)	0.0297 (10)	0.0420 (12)	0.0323 (12)
N32	0.0481 (9)	0.0467 (9)	0.0694 (11)	0.0156 (7)	0.0258 (8)	0.0156 (8)
O21	0.0544 (8)	0.0479 (7)	0.0498 (8)	0.0116 (6)	0.0047 (6)	0.0080 (6)
O22	0.0483 (7)	0.0390 (7)	0.0474 (8)	0.0021 (6)	0.0062 (6)	0.0068 (6)
O31	0.0769 (11)	0.0799 (11)	0.0826 (12)	0.0274 (9)	0.0238 (10)	0.0229 (10)
O32	0.0594 (8)	0.0453 (8)	0.0695 (10)	0.0120 (6)	0.0191 (7)	0.0126 (7)

### Geometric parameters (Å, °)

**Table:**

O1W—Na	2.4117 (17)	C22—N22	1.290 (2)
O1W—Na^i^	2.4851 (18)	C22—C23	1.458 (2)
O1W—H1WA	0.80 (4)	C23—C24	1.379 (3)
O1W—H1WB	0.89 (4)	C23—C28	1.409 (2)
O2W—Na	2.361 (2)	C24—C25	1.389 (3)
O2W—H2WB	0.90 (3)	C24—H24	0.9300
O2W—H2WA	0.92 (4)	C25—C26	1.382 (3)
Na—O11^ii^	2.3281 (14)	C25—H25	0.9300
Na—O1W^i^	2.4851 (18)	C26—C27	1.382 (3)
Na—N12	2.4906 (15)	C26—H26	0.9300
Na—O11	2.5721 (14)	C27—C28	1.374 (3)
Na—Na^i^	3.7874 (13)	C27—H27	0.9300
Na—Na^ii^	3.8590 (13)	C28—N21	1.400 (2)
C11—O11	1.224 (2)	C31—O31	1.223 (3)
C11—N11	1.354 (2)	C31—N31	1.349 (3)
C11—C12	1.497 (2)	C31—C32	1.508 (3)
C12—N12	1.2954 (19)	C32—N32	1.288 (2)
C12—C13	1.450 (2)	C32—C33	1.450 (3)
C13—C14	1.387 (2)	C33—C34	1.386 (3)
C13—C18	1.403 (2)	C33—C38	1.405 (3)
C14—C15	1.384 (2)	C34—C35	1.396 (3)
C14—H14	0.9300	C34—H34	0.9300
C15—C16	1.388 (3)	C35—C36	1.393 (4)
C15—H15	0.9300	C35—H35	0.9300
C16—C17	1.380 (2)	C36—C37	1.382 (4)
C16—H16	0.9300	C36—H36	0.9300
C17—C18	1.379 (2)	C37—C38	1.370 (3)
C17—H17	0.9300	C37—H37	0.9300
C18—N11	1.403 (2)	C38—N31	1.418 (3)
N11—H11	0.92 (2)	N21—H21	0.88 (2)
N12—O12	1.3598 (16)	N22—O22	1.3730 (19)
O11—Na^ii^	2.3281 (14)	N31—H31	0.92 (2)
C21—O21	1.233 (2)	N32—O32	1.380 (2)
C21—N21	1.357 (2)	O22—H22	0.94 (2)
C21—C22	1.498 (2)	O32—H32	1.01 (3)
			
Na—O1W—Na^i^	101.31 (6)	C12—N12—O12	114.48 (13)
Na—O1W—H1WA	108 (3)	C12—N12—Na	115.23 (10)
Na^i^—O1W—H1WA	112 (3)	O12—N12—Na	130.29 (9)
Na—O1W—H1WB	118 (2)	C11—O11—Na^ii^	143.47 (12)
Na^i^—O1W—H1WB	106 (3)	C11—O11—Na	110.20 (10)
H1WA—O1W—H1WB	111 (3)	Na^ii^—O11—Na	103.80 (5)
Na—O2W—H2WB	114.6 (19)	O21—C21—N21	126.21 (17)
Na—O2W—H2WA	130 (3)	O21—C21—C22	128.03 (16)
H2WB—O2W—H2WA	109 (3)	N21—C21—C22	105.76 (15)
O11^ii^—Na—O2W	87.67 (8)	N22—C22—C23	134.90 (16)
O11^ii^—Na—O1W	96.19 (7)	N22—C22—C21	118.13 (16)
O2W—Na—O1W	162.92 (8)	C23—C22—C21	106.95 (14)
O11^ii^—Na—O1W^i^	114.02 (7)	C24—C23—C28	119.79 (16)
O2W—Na—O1W^i^	84.53 (8)	C24—C23—C22	134.28 (16)
O1W—Na—O1W^i^	78.69 (6)	C28—C23—C22	105.92 (15)
O11^ii^—Na—N12	141.63 (5)	C23—C24—C25	118.61 (18)
O2W—Na—N12	99.06 (8)	C23—C24—H24	120.7
O1W—Na—N12	88.24 (7)	C25—C24—H24	120.7
O1W^i^—Na—N12	104.24 (7)	C26—C25—C24	120.6 (2)
O11^ii^—Na—O11	76.20 (5)	C26—C25—H25	119.7
O2W—Na—O11	77.10 (7)	C24—C25—H25	119.7
O1W—Na—O11	119.99 (6)	C27—C26—C25	121.82 (19)
O1W^i^—Na—O11	158.74 (6)	C27—C26—H26	119.1
N12—Na—O11	68.78 (4)	C25—C26—H26	119.1
O11^ii^—Na—Na^i^	109.62 (5)	C28—C27—C26	117.46 (18)
O2W—Na—Na^i^	123.11 (7)	C28—C27—H27	121.3
O1W—Na—Na^i^	40.05 (4)	C26—C27—H27	121.3
O1W^i^—Na—Na^i^	38.64 (4)	C27—C28—N21	128.73 (17)
N12—Na—Na^i^	98.16 (4)	C27—C28—C23	121.74 (18)
O11—Na—Na^i^	158.40 (5)	N21—C28—C23	109.52 (15)
O11^ii^—Na—Na^ii^	40.34 (3)	O31—C31—N31	126.3 (2)
O2W—Na—Na^ii^	80.01 (7)	O31—C31—C32	127.9 (2)
O1W—Na—Na^ii^	113.47 (5)	N31—C31—C32	105.8 (2)
O1W^i^—Na—Na^ii^	150.08 (7)	N32—C32—C33	135.3 (2)
N12—Na—Na^ii^	103.40 (4)	N32—C32—C31	117.4 (2)
O11—Na—Na^ii^	35.87 (3)	C33—C32—C31	107.33 (17)
Na^i^—Na—Na^ii^	145.31 (4)	C34—C33—C38	120.1 (2)
O11—C11—N11	126.48 (15)	C34—C33—C32	134.14 (19)
O11—C11—C12	127.36 (14)	C38—C33—C32	105.8 (2)
N11—C11—C12	106.15 (14)	C33—C34—C35	118.6 (2)
N12—C12—C13	134.80 (14)	C33—C34—H34	120.7
N12—C12—C11	118.35 (14)	C35—C34—H34	120.7
C13—C12—C11	106.84 (12)	C36—C35—C34	119.7 (3)
C14—C13—C18	119.60 (14)	C36—C35—H35	120.2
C14—C13—C12	134.32 (14)	C34—C35—H35	120.2
C18—C13—C12	106.07 (13)	C37—C36—C35	122.3 (2)
C15—C14—C13	118.58 (15)	C37—C36—H36	118.9
C15—C14—H14	120.7	C35—C36—H36	118.9
C13—C14—H14	120.7	C38—C37—C36	117.5 (3)
C14—C15—C16	120.90 (16)	C38—C37—H37	121.3
C14—C15—H15	119.5	C36—C37—H37	121.3
C16—C15—H15	119.5	C37—C38—C33	121.9 (3)
C17—C16—C15	121.37 (16)	C37—C38—N31	128.3 (2)
C17—C16—H16	119.3	C33—C38—N31	109.82 (19)
C15—C16—H16	119.3	C21—N21—C28	111.79 (15)
C18—C17—C16	117.64 (15)	C21—N21—H21	122.1 (14)
C18—C17—H17	121.2	C28—N21—H21	126.1 (14)
C16—C17—H17	121.2	C22—N22—O22	111.60 (14)
C17—C18—C13	121.90 (15)	C31—N31—C38	111.2 (2)
C17—C18—N11	128.29 (14)	C31—N31—H31	117.4 (15)
C13—C18—N11	109.79 (13)	C38—N31—H31	130.6 (15)
C11—N11—C18	111.13 (13)	C32—N32—O32	112.35 (17)
C11—N11—H11	122.0 (13)	N22—O22—H22	102.9 (14)
C18—N11—H11	126.9 (13)	N32—O32—H32	100.8 (16)
			
Na^i^—O1W—Na—O11^ii^	113.32 (8)	Na^ii^—Na—O11—C11	166.41 (17)
Na^i^—O1W—Na—O2W	11.0 (4)	O11^ii^—Na—O11—Na^ii^	0.0
Na^i^—O1W—Na—O1W^i^	0.0	O2W—Na—O11—Na^ii^	90.76 (9)
Na^i^—O1W—Na—N12	−104.90 (8)	O1W—Na—O11—Na^ii^	−89.23 (9)
Na^i^—O1W—Na—O11	−169.06 (6)	O1W^i^—Na—O11—Na^ii^	121.64 (19)
Na^i^—O1W—Na—Na^ii^	151.25 (6)	N12—Na—O11—Na^ii^	−164.04 (8)
O11—C11—C12—N12	1.2 (3)	Na^i^—Na—O11—Na^ii^	−108.60 (12)
N11—C11—C12—N12	−179.47 (14)	O21—C21—C22—N22	−4.4 (3)
O11—C11—C12—C13	−179.19 (18)	N21—C21—C22—N22	176.21 (14)
N11—C11—C12—C13	0.14 (18)	O21—C21—C22—C23	177.07 (17)
N12—C12—C13—C14	−0.9 (3)	N21—C21—C22—C23	−2.36 (17)
C11—C12—C13—C14	179.58 (18)	N22—C22—C23—C24	2.9 (3)
N12—C12—C13—C18	178.88 (17)	C21—C22—C23—C24	−178.91 (18)
C11—C12—C13—C18	−0.64 (17)	N22—C22—C23—C28	−176.34 (18)
C18—C13—C14—C15	0.2 (2)	C21—C22—C23—C28	1.88 (17)
C12—C13—C14—C15	179.93 (17)	C28—C23—C24—C25	−0.4 (2)
C13—C14—C15—C16	0.2 (3)	C22—C23—C24—C25	−179.56 (17)
C14—C15—C16—C17	−0.2 (3)	C23—C24—C25—C26	−0.2 (3)
C15—C16—C17—C18	−0.1 (3)	C24—C25—C26—C27	0.8 (3)
C16—C17—C18—C13	0.5 (3)	C25—C26—C27—C28	−0.6 (3)
C16—C17—C18—N11	178.98 (17)	C26—C27—C28—N21	−179.25 (17)
C14—C13—C18—C17	−0.5 (2)	C26—C27—C28—C23	−0.1 (3)
C12—C13—C18—C17	179.66 (15)	C24—C23—C28—C27	0.6 (2)
C14—C13—C18—N11	−179.26 (14)	C22—C23—C28—C27	179.98 (15)
C12—C13—C18—N11	0.91 (17)	C24—C23—C28—N21	179.90 (15)
O11—C11—N11—C18	179.77 (18)	C22—C23—C28—N21	−0.75 (18)
C12—C11—N11—C18	0.43 (19)	O31—C31—C32—N32	4.0 (3)
C17—C18—N11—C11	−179.51 (17)	N31—C31—C32—N32	−176.08 (16)
C13—C18—N11—C11	−0.87 (19)	O31—C31—C32—C33	−177.3 (2)
C13—C12—N12—O12	2.1 (3)	N31—C31—C32—C33	2.7 (2)
C11—C12—N12—O12	−178.47 (13)	N32—C32—C33—C34	−3.9 (4)
C13—C12—N12—Na	−178.03 (15)	C31—C32—C33—C34	177.7 (2)
C11—C12—N12—Na	1.44 (18)	N32—C32—C33—C38	176.3 (2)
O11^ii^—Na—N12—C12	−27.44 (16)	C31—C32—C33—C38	−2.16 (19)
O2W—Na—N12—C12	70.32 (13)	C38—C33—C34—C35	0.3 (3)
O1W—Na—N12—C12	−125.20 (12)	C32—C33—C34—C35	−179.50 (19)
O1W^i^—Na—N12—C12	156.94 (11)	C33—C34—C35—C36	−0.4 (3)
O11—Na—N12—C12	−1.95 (11)	C34—C35—C36—C37	0.0 (3)
Na^i^—Na—N12—C12	−164.11 (11)	C35—C36—C37—C38	0.5 (3)
Na^ii^—Na—N12—C12	−11.48 (12)	C36—C37—C38—C33	−0.6 (3)
O11^ii^—Na—N12—O12	152.45 (12)	C36—C37—C38—N31	178.4 (2)
O2W—Na—N12—O12	−109.79 (13)	C34—C33—C38—C37	0.2 (3)
O1W—Na—N12—O12	54.69 (13)	C32—C33—C38—C37	−179.93 (18)
O1W^i^—Na—N12—O12	−23.17 (13)	C34—C33—C38—N31	−178.94 (17)
O11—Na—N12—O12	177.94 (13)	C32—C33—C38—N31	0.9 (2)
Na^i^—Na—N12—O12	15.78 (13)	O21—C21—N21—C28	−177.48 (16)
Na^ii^—Na—N12—O12	168.41 (11)	C22—C21—N21—C28	1.97 (18)
N11—C11—O11—Na^ii^	−24.6 (4)	C27—C28—N21—C21	178.38 (17)
C12—C11—O11—Na^ii^	154.56 (15)	C23—C28—N21—C21	−0.82 (19)
N11—C11—O11—Na	177.89 (15)	C23—C22—N22—O22	−2.8 (3)
C12—C11—O11—Na	−2.9 (2)	C21—C22—N22—O22	179.10 (13)
O11^ii^—Na—O11—C11	166.41 (17)	O31—C31—N31—C38	177.8 (2)
O2W—Na—O11—C11	−102.83 (15)	C32—C31—N31—C38	−2.1 (2)
O1W—Na—O11—C11	77.18 (16)	C37—C38—N31—C31	−178.2 (2)
O1W^i^—Na—O11—C11	−71.9 (2)	C33—C38—N31—C31	0.8 (2)
N12—Na—O11—C11	2.38 (13)	C33—C32—N32—O32	2.5 (3)
Na^i^—Na—O11—C11	57.8 (2)	C31—C32—N32—O32	−179.19 (15)

Symmetry codes: (i) −*x*, −*y*+1, −*z*; (ii) −*x*+1, −*y*+1, −*z*.

### Hydrogen-bond geometry (Å, °)

**Table:**

*D*—H···*A*	*D*—H	H···*A*	*D*···*A*	*D*—H···*A*
N21—H21···O21^iii^	0.87 (2)	1.97 (2)	2.846 (5)	175 (2)
O22—H22···O12	0.94 (2)	1.70 (2)	2.633 (3)	171 (2)
O22—H22···N12	0.94 (2)	2.45 (2)	3.226 (4)	140 (2)

Symmetry codes: (iii) −*x*−1, −*y*, −*z*.
